# Diet induced obesity alters muscle spindle afferent function in adult mice

**DOI:** 10.1371/journal.pone.0196832

**Published:** 2018-05-02

**Authors:** Lubayna S. Elahi, Krystle N. Shamai, Adam M. Abtahie, Adam M. Cai, Shreejit Padmanabhan, Martina Bremer, Katherine A. Wilkinson

**Affiliations:** 1 Department of Biological Sciences, San José State University, San Jose, California, United States of America; 2 Department of Mathematics & Statistics San José State University, San Jose, California, United States of America; New York Medical College, UNITED STATES

## Abstract

Populations with obesity are more likely to fall and exhibit balance instability. The reason for this is likely multifactorial, but there is some evidence that sensory function is impaired during obesity. We tested the hypothesis that muscle proprioceptor function is compromised in a mouse model of diet induced obesity. An *in vitro* muscle-nerve preparation was used to record muscle spindle afferent responses to physiological stretch and sinusoidal vibration. We compared the responses of C57/Bl6 male and female mice on a control diet (10% kcal fat) with those eating a high fat diet (HFD; 60% kcal fat) for 10 weeks (final age 14–15 weeks old). Following HFD feeding, adult mice of both sexes exhibited decreased muscle spindle afferent responses to muscle movement. Muscle spindle afferent firing rates during the plateau phase of stretch were significantly lower in both male and female HFD animals as were two measures of dynamic sensitivity (dynamic peak and dynamic index). Muscle spindle afferents in male mice on a HFD were also significantly less likely to entrain to vibration. Due to the importance of muscle spindle afferents to proprioception and motor control, decreased muscle spindle afferent responsiveness may contribute to balance instability during obesity.

## Introduction

Populations with obesity are more likely to fall [1–3], more likely to visit the hospital with fall related injuries [4], and have a higher risk of disability from falls than normal weight populations [5]. Populations with obesity also exhibit increased sway during standing and altered gait [6–9], which are risk factors for falling [10] and suggestive of proprioceptive deficits. The reason for impaired balance in obesity is not completely understood. The increase and change in center of mass during obesity is important [11–13], but does not appear to be the only contributor [14]. Higher variability in balance motor commands is observed in people with obesity that could be due to less reliable sensory information and/or increased reliance on the more variable input from the visual and vestibular systems [15]. People with obesity also exhibit a decreased ability to use somatosensory input to maintain balance [16], a decreased ability to discriminate between object weights [17], and require more attentional skills to maintain posture in difficult postural conditions [18]. Decreased fine motor skills are also observed in children with obesity even when sitting [19]. These findings suggest that obesity may alter the central processing and/or function of somatosensory afferents, but few studies have directly tested this hypothesis.

A better understanding of how obesity affects somatosensory afferents is needed due to their importance in motor control and the maintenance of balance. The sensitivity of one class of somatosensory afferents, the plantar mechanoreceptors that provide information about foot pressure and placement [20], is decreased in people with obesity [21–23]. The primary proprioceptive afferents are the Group Ia and II sensory neurons that innervate the muscle spindle [24] and the effect of obesity on muscle spindle afferents is unknown. Muscle spindle afferents convey information about muscle length and limb position to the central nervous system which is then used to develop a three dimensional representation of body position in space [24, 25]. In addition, the Group Ia muscle spindle afferents also comprise the sensory component of the monosynaptic muscle stretch reflex, which is critical for fast error correction during ongoing movement [24]. Impairment in muscle spindle afferent structure and function is observed in other conditions accompanied by poor balance and an increased risk of falling, including aging and diabetes [26–30].

Direct measurement of sensory afferent response properties in humans is difficult, as is properly controlling for all co-morbidities, including diabetic neuropathy, in populations with obesity. In this study we used a mouse model of high fat diet induced obesity to study obesity-related sensory impairments. Diet induced obesity in mice leads to altered balance as evidenced by a reduction in motor coordination on the rotorod test, especially when the speed is varied, and increased slipping during a beam walking test [31–33]. Diet induced obesity also leads to alterations in gait [32] and decreases voluntary locomotor speed, which is similar to the decreased gait speed observed in humans [31, 34]. We tested the hypothesis that muscle spindle afferent function is altered in a mouse model of diet induced obesity. We used an *in vitro* muscle-nerve preparation to measure muscle spindle afferent responses to physiological stretch and vibration in adult mice of both sexes [35]. Male mice are known to exhibit greater inflammatory and metabolic changes [36] as well as greater [37] and faster weight gain [38] than female mice when fed a high fat diet, so we also determined whether there was a sex difference in the muscle spindle afferent response to diet induced obesity.

## Materials and methods

### Animals and diets

All of the procedures were approved and authorized by the Institutional Animal Care and Use Committee at San José State University (Protocol #1001). Forty-four C57BL/6 4–5 wk old mice (22 M, 22 F) were purchased from Simonsen Laboratories (Gilroy, CA) and housed in cages of 5–8 mice under a 12:12 hour light-dark cycle. All mice were fed the control diet (10% kcal fat; D12450J Research Diets; New Brunswick, NJ) for a period of 1 week to acclimatize them to the texture of the special diet. After 1 week the mice were assigned to one of 2 experimental conditions for the following 10 weeks. The control group (CON) remained on the control diet (n = 10 M and 11 F) while the high fat diet group (HFD, n = 12 M and 11 F) was fed a high fat diet (60% kcal fat; Research Diets D12492). Both diets contain 20% kcal from protein, with the remaining kcal coming from carbohydrates (extra carbohydrates in CON diet from corn starch, extra fat in HFD from lard). Visual health and activity checks were performed daily by trained animal care staff and cages changed twice a week. Animals were weighed once a week for the duration of the study.

### Electrophysiological recording of muscle sensory neuron activity

Direct recording of muscle sensory neuron function was performed using an isolated extensor digitorum longus (EDL) muscle-nerve preparation. The EDL was chosen because it has accessible tendons, is thin enough to allow adequate oxygen diffusion [39], and we have previously characterized control responses to stretch and vibration of EDL muscle spindle afferents [35]. Detailed methods can be found in [40]. On the day of the experiment mice were placed in an induction chamber, deeply anesthetized with isoflurane (5%), decapitated, and skinned. The legs were removed and placed into an oxygenated (95% O_2_/5% CO_2_) dish filled with low calcium, high magnesium artificial cerebrospinal fluid, containing in mM: 128 NaCl, 1.9 KCl, 1.2 KH_2_PO_4_, 26 NaHCO_3_, 0.85 CaCl_2_, 6.5 MgSO_4_, and 10 glucose (pH of 7.4). The EDL muscle and the deep peroneal branch of the sciatic nerve were dissected and placed in an oxygenated (100% O_2_) tissue bath of synthetic interstitial fluid containing in mM 123 NaCl, 3.5 KCl, 0.7 MgSO_4_, 1.7 NaH_2_PO_4_, 2.0 CaCl_2_, 9.5 NaC_6_H_11_O (sodium gluconate), 5.5 glucose, 7.5 sucrose, and 10 *N*-2-hydroxyethylpiperazine-*N′*-2-ethanesulfonic acid (HEPES) (pH 7.4±0.05; [41]). Both tendons were sutured with 5–0 nylon thread, with one end tied to a fixed post and the other end tied to the lever arm of a dual force and length controller and transducer (300C-LR, Aurora Scientific, Inc.; Aurora, ON, Canada). All experiments were conducted at 24°C, conditions found to maintain tissue quality for extended periods of time and to produce muscle spindle afferent responses similar to those at body temperature [35].

The length at which the muscle produced the maximal twitch contractile force, or optimal length (L_o_), was determined following stimulation via bath electrodes (0.5 ms pulse width, supramaximal voltage; S88 Stimulator, Grass Technologies; San Carlos, CA). The cut end of the nerve was suctioned into a bipolar glass electrode and connected to an extracellular amplifier with headstage (Model 1800, A-M Systems; Sequim, WA). Neural responses to a battery of ramp and hold stretches and sinusoidal vibrations were then digitized and recorded (PowerLab, ADInstruments; Sydney, Australia). The muscle was stretched to three physiological lengths (2.5%, 5%, and 7.5% of L_o_; 40% L_o_/s ramp speed). Each stretch was repeated three times with a 1 min rest period in between to prevent muscle thixotropic effects. Sinusoidal vibrations 9 s in length were performed at 4 frequencies (10, 25, 50, and 100 Hz) and 4 amplitudes (5, 25, 50 and 100 μm) with 1 min rest in between, for a total of 16 different vibrations. At the end of the experiment the muscle was contracted 60 times at 1 Hz frequency (0.5 ms pulse width). The maximal tetanic contractile force was then measured (500 ms train, 120 Hz, 0.5 ms pulse width). Wet weight of the EDL was determined and cross sectional area (CSA) calculated as (mass)/(L_o_ x density), using 1.06 kg/L for muscle density [42, 43].

### Data analysis

**Fig 1:**
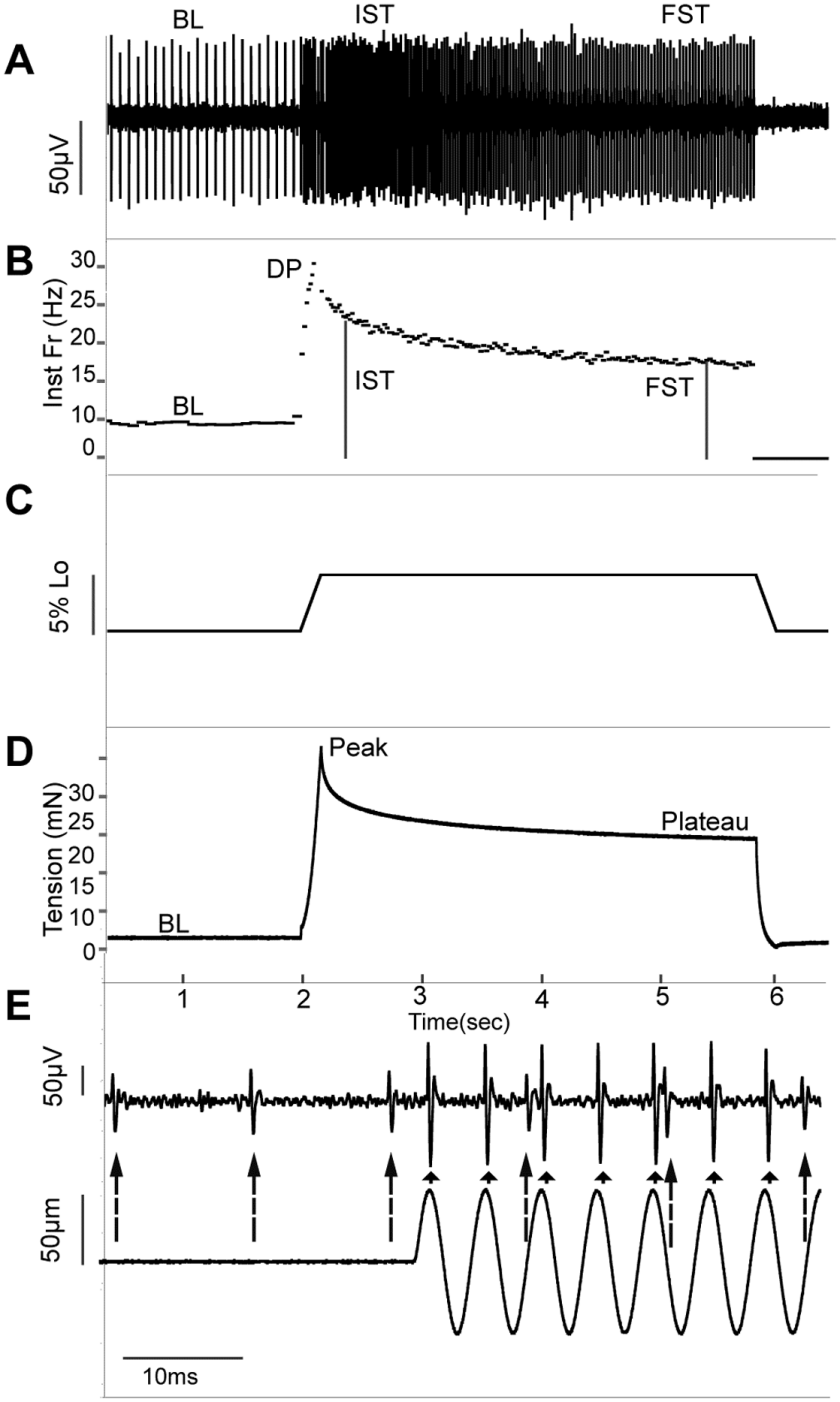
Data analysis procedure. **(A)** Raw trace of muscle spindle afferent response to ramp and hold stretch. Times where baseline (BL), initial static time (IST), and final static time (FST) firing frequencies measured are shown on the trace. **(B)** Instantaneous frequency (inst fr; Hz) response to ramp and hold stretch of identified unit during stretch. Time BL, dynamic peak (DP), IST, and FST measured identified on graph. **(C)** Length change during stretch. **(D)** Muscle tension during stretch. Time where baseline tension (BL), maximum tension (peak), and end of ramp tension (plateau) were measured indicated on trace. **(E)** Raw trace of neural response (top) to sinusoidal vibration (length change bottom). The larger unit exhibits 1:1 entrainment as denoted by small black arrows. The smaller unit’s firing rate is unaffected by vibration as denoted by large dashed arrows.

The Spike Histogram function of Lab Chart (ADInstruments) was used to identify individual afferents based on spike shape. Individual muscle spindle afferents were identified functionally by determining if they increased firing frequency to stretch and paused during the 60 twitch contractions [44]. A total of 78 muscle spindle afferents were used in this study (17 M CON from 14 muscles, 21 M HFD from 19 muscles, 20 F CON from 14 muscles, 20 F HFD from 16 muscles). Due to technical reasons, responses to both stretch and vibration were not recorded for all afferents and the numbers used for each test are denoted in the relevant figure. Instantaneous firing frequency was measured at baseline (BL), at the beginning of stretch (0.5 s after ramp completed, initial static time or IST), and during the plateau phase of stretch (0.5s before end of stretch, final static time or FST). The peak firing during the ramp phase was also measured (dynamic peak or DP; Fig 1A & 1B). Values were averaged for the 3 repeats of each stretch as no systematic order effect was observed. Dynamic Index (DI) was calculated as DP-IST. For each vibration we determined if the afferent could entrain by firing at the same time during each cycle of vibration (Fig 1E). We confirmed that all afferents with BL firing paused in response to twitch contraction, with the exception of 13 afferents that could not be analyzed due to technical issues. We are reasonably confident that only muscle spindle afferents were included in our sample because all included afferents displayed the characteristic adaptation of firing frequency during the hold phase of stretch typical of muscle spindle afferents (Fig 1B). Group Ib Golgi Tendon Organ afferents rarely discharge to stretches of the lengths given [45] and would have increased firing rate during contraction, something we never observed. Similarly, Group III/IV afferents typically do not respond to physiological stretch and would not be expected to pause during twitch contraction [46].

Maximal tetanic contraction strength normalized to muscle CSA was determined for each muscle. Muscle tension at baseline, peak tension during stretch, and plateau tension immediately before stretch was released were determined at the 5% L_o_ stretch length (Fig 1D). We calculated the parallel (E_PE_) and series modulus of elasticity (E_SE_) normalized to muscle CSA using the following formula from [43]:

$$E=\frac{\frac{\Delta F}{CSA}}{\frac{\Delta L}{L_{o}}}$$

#### Equation 1: Modulus of elasticity

Δ*F* is the difference in tension (mN) from baseline; Δ*L* is the change in length (mm) from L_o_. Δ*F* measured from baseline to peak of stretch for E_SE_ and baseline to end of stretch for E_PE_.

### Statistics

Body weight changes over the course of experiment and body weights the day of experiment were compared using independent samples t-tests to the CON value of the same sex. Weekly body weights were also compared using independent sample t-tests to the CON value of the same sex followed by a Hochberg correction. The proportions of afferents without baseline firing were compared with a two sample z-test. Group BL, DP, IST, FST, and DI were compared using a three factor ANOVA model, with stretch length, sex, and diet as factors. Muscle weight, L_o_, CSA, BL muscle tension, peak muscle tension, plateau muscle tension, E_PE_, E_SE_, and maximal tetanic contraction strength were compared with a two factor ANOVA model (sex and diet). Ability to entrain to vibration was fitted as a function of vibration amplitude, vibration frequency, sex, and diet using a logistic regression model. As we found a sex*diet interaction, we fit a logistic regression model for each sex as a function of vibration frequency, vibration amplitude, and diet. Values are given as mean ± standard deviation, error bars on graphs indicate 95% confidence intervals, and all differences are considered significant if p<0.05.

## Results

### Weight gain

**Fig 2:**
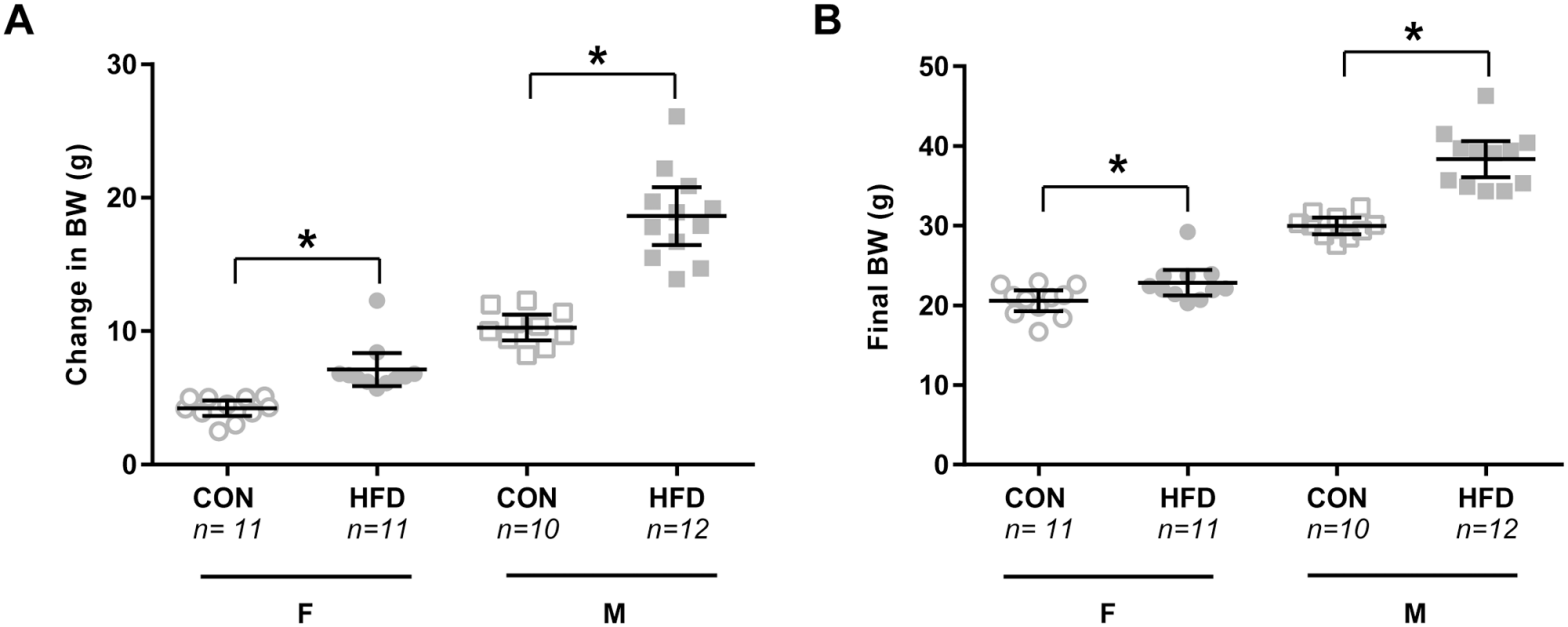
Weight gain over 10-week treatment period. Change in body weight over the 10-week treatment period (**A**) and final body weights (**B**) shown for each individual animal (circles for F, squares for M) as well as group means and 95% confidence interval. *p<0.05 versus same sex CON; independent samples t-test.

Mice of both sexes on a HFD gained more weight after 10 weeks of treatment as compared to CON mice of the same sex (both M & F p<0.001; Fig 2A). On average M HFD mice weighed ~28% more (p<0.001) and F HFD mice ~10% more than CON (p = 0.025; Fig 2B) on the day of experiment, which would qualify as a moderate level of obesity [47]. M HFD mice were significantly heavier than their CON diet counterparts starting at week 5, whereas F HFD mice were only significantly heavier in the last week.

### Muscle spindle afferent response to movement altered by HFD

**Fig 3:**
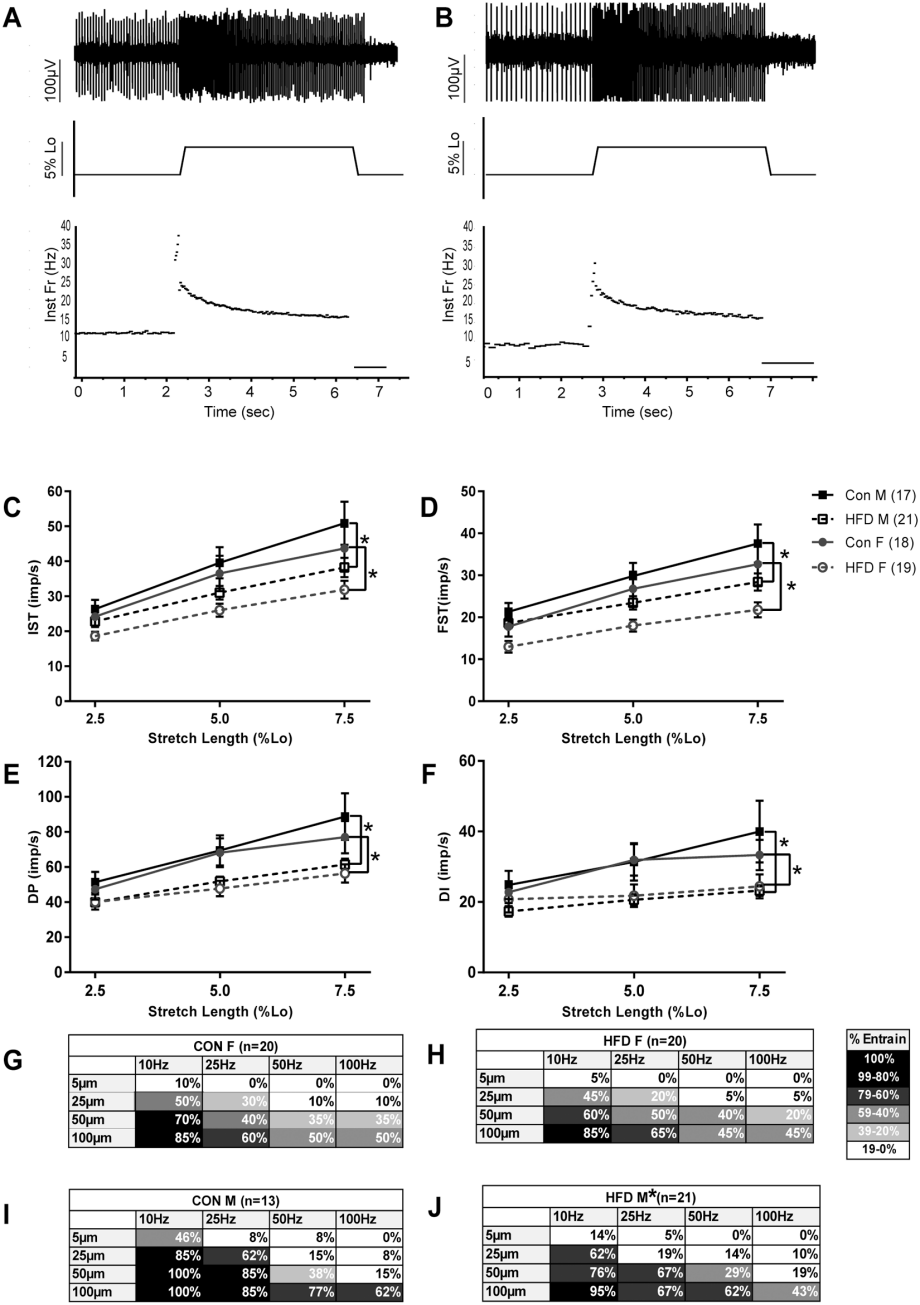
Mice fed a HFD exhibit impaired muscle spindle afferent responses to stretch and vibration. Example raw and instantaneous frequency traces from a representative M CON (**A**) and M HFD (**B**) animal. Firing rates 0.5s (IST; **C**) and 3.5s (FST; **D**) into stretch were significantly decreased in both M and F mice fed a HFD. F mice had significantly lower IST and FST responses than M mice. Measures of dynamic responsiveness to stretch were also lower in HFD mice of both sexes (DP **E**; DI **F**) and there was no sex difference. Error bars denote ± SEM; *denotes p<0.05 from CON of same sex using three factor ANOVA (stretch length, diet, sex). Tables **G-J** denote percentage of afferents that can entrain to each of the 16 vibrations. Vibration amplitude increases from top to bottom and frequency increases left to right. Darker shades denote a greater percentage of entrained units. Overall, HFD animals less likely to entrain to vibration, though no difference in ability to entrain to vibration was observed between CON and HFD female mice when analyzed separately using a logistic regression model (amplitude, frequency, diet; **G & H**). Afferents from male mice fed a HFD (**J**) less likely to entrain to vibration than M CON afferents (**I**). *p<0.05 from M CON.

We first tested the hypothesis that 10 weeks on a HFD would alter muscle spindle afferent static sensitivity in both male and female mice. We compared muscle spindle afferent firing rates at BL, 0.5 s into stretch (IST), and 3.5 s into stretch (FST). Overall, mice fed a HFD were less likely to have a non-zero BL firing rate (71% CON vs 54% HFD; p<0.05), however there was a sex difference in this response. In M mice, significantly more CON afferents had a non-zero BL firing rate (88% M CON vs. 52% M HFD; p<0.05) and the CON proportion was similar to that observed in our previous study [35]. In F mice, fewer CON afferents had BL firing than in the male CON group, but there was no significant difference in the percentage of afferents with BL firing between conditions (53% F CON vs. 56% F HFD; p = 0.87). Average BL firing rates of afferents with non-zero firing rates was significantly lower in F mice than M mice (F BL: 9.0 ± 4.4 Hz vs. M BL: 12.5 ± 5.4 Hz; sex main effect p<0.05), but BL firing rate was not significantly different with HFD (diet main effect p = 0.26; sex * diet p = 0.63). Both male and female CON and HFD mice showed linear increases in firing rate at both the beginning (IST) and end (FST) of stretch in response to increasing stretch length as expected (stretch length main effect p<0.001 for IST and FST). IST and FST were significantly lower in HFD mice than CON mice (diet main effect p<0.001 for IST and FST), with both M and F mice exhibiting the same pattern (sex * diet p = 0.90 for IST and p = 0.63 for FST). There was also a sex difference, with F mice having lower IST and FST firing rates than M mice (sex main effect p = 0.024 for IST and p<0.01 FST; Fig 3A–3D). Overall, static sensitivity of muscle spindle afferents following HFD was lower in both M and F mice.

We next tested the hypothesis that muscle spindle afferent dynamic sensitivity was altered by HFD in mice of both sexes by measuring DP, DI, and the ability to entrain to vibration. DP, or the peak firing frequency during the ramp phase of stretch, was significantly lower in HFD mice of both sexes (main effect diet p<0.001; main effect stretch length p<0.001; main effect sex p = 0.25; sex*diet p = 0.73; Fig 3E). Similarly, DI was significantly lower in HFD animals of both sexes (main effect diet p<0.001; main effect stretch length p<0.01; main effect sex p = 0.87; sex*diet p = 0.32; Fig 3F). As expected, afferents were less able to entrain to low amplitude and high frequency vibrations (main effect of amplitude & vibration p<0.001). On average, HFD afferents were less able to entrain to vibration, especially low amplitude vibration, than CON afferents, suggesting decreased dynamic sensitivity in HFD mice (main effect diet p = 0.01; Fig 3G–3J). However, there was a sex difference as well (main effect of sex p<0.001). There was a significant effect of HFD when M mice alone were analyzed (main effect diet p = 0.04) but not when F mice alone were analyzed (main effect diet p = 0.45). Overall, dynamic sensitivity was reduced following a HFD.

### Muscle elasticity and contractility unchanged by HFD

EDL muscle wet weight, L_o_, and CSA were not altered by HFD (main effect of diet p = 0.31, 0.29, 0.75 respectively). As expected due to the lower female body weights, EDL muscle weight and L_o_, but not CSA were significantly lower in female mice (main effect of sex p<0.001 for muscle weight, p<0.05 for L_o_, p = 0.13 for CSA; Table 1). Maximum tetanic contractile force was not significantly different among groups and all groups were in the range of previously reported values for healthy EDL muscles (20.6 ± 1.0 N/m^2^; [42]). Muscle parallel (E_PE_) and series (E_SE_) elasticity were unchanged for M and F mice on a HFD compared to CON (diet: E_PE_ p = 0.52 & E_SE_ p = 0.40; sex*diet E_PE_ p = 0.24 & E_SE_ p = 0.30; Table 1). Similarly, BL, peak, and plateau tensions were unchanged with HFD (diet: BL p = 0.68, peak p = 0.15, plateau p = 0.24; sex*diet: BL p = 0.17, peak p = 0.94, plateau p = 0.30; Table 1). However, F mice had significantly lower tension and elasticity values when compared to M (p<0.001 for all measures; Table 1). Overall, HFD treatment did not alter any of the muscle anatomical or functional properties measured.

**Table 1:** Muscle properties unchanged by HFD.

Condition	Muscle Weight (mg)	L_0_ (mm)	CSA (mm^2^)	BL Tension (mN)	Peak Tension (mN)	Plateau Tension (mN)	E_SE_ (MPa)	E_PE_ (MPa)
**M CON (*n* = 14)**	11.48 ± 1.27	11.81 ± 1.14	0.93 ± 0.14	6.14 ± 1.67	51.60 ± 17.21	30.52 ± 10.19	1.81 ± 0.75	1.21 ± 0.48
**M HFD (*n* = 19)**	10.61 ± 1.68	11.90 ± 1.21	0.85 ± 0.13	5.36 ± 1.77	46.31 ± 12.69	26.93 ± 7.80	1.84 ± 0.67	1.27 ± 0.42
**F CON (*n* = 14)**	9.44 ± 1.41	11.35 ± 1.23	0.80 ± 0.18	3.67 ± 1.56	30.60 ± 15.04	16.68 ± 7.48	1.27 ± 0.66	0.91 ± 0.48
**F HFD (*n* = 16)**	9.52 ± 1.65	10.60± 1.28	0.86 ± 0.16	4.09 ± 1.73	25.84 ± 8.29	15.49 ± 6.23	0.97 ± 0.39	0.71 ± 0.27

No differences in muscle size, tension during any point of stretch, or muscle elasticity observed following high fat feeding. Values of all measures except CSA significantly lower in F mice than M mice. Values compared using two factor ANOVA (diet, sex). Group averages shown ± standard deviation.

## Discussion

### Muscle proprioceptor function impaired in a mouse model of diet induced obesity

Balance instability and an increased rate of falling are seen in human populations with obesity of all ages and both sexes [1, 3, 9]. Similarly, mice fed a high fat diet also exhibit balance and gait impairments [31–33]. In this study we identified a possible contributing factor: impairment in muscle proprioceptor function. Muscle spindle afferent receptor endings were less responsive to both muscle stretch and sinusoidal vibration in adult mice fed a HFD for 10 wks. Both static (Fig 3A–3D) and dynamic muscle spindle afferent sensitivity (Fig 3E–3J) were decreased, suggesting that the central nervous system receives inaccurate muscle position and movement information following diet induced obesity.

Muscle spindle afferent function is critical to representing body position in space as well as fast error correction via the muscle stretch reflex [24]. We measured muscle spindle afferent function during passive conditions without gamma motor neuron tone or other central nervous system input. Our results suggest that muscle spindle input to alpha motor neurons is lower during obesity, potentially decreasing muscle tone. Similarly, our results suggest that muscle stretch reflex strength would be lower in obese animals unless obesity also leads to increased gamma motor neuron tone and/or central reflex excitability. Future studies are necessary to determine the effect on muscle tone and motor behavior from these observed changes in passive mechanoreception. Future studies could also address if longer treatment with a HFD will lead to more severe changes in muscle spindle afferent function or whether exposure at different developmental stages would change our results.

Alterations in muscle spindle afferent function occur in two other conditions associated with impaired balance: aging [26, 29, 48] and diabetes [27, 30]. In particular, aged rats exhibit decreased firing rates during static stretch and decreased dynamic sensitivity [29], similar to what we have observed. Due to the important role of muscle spindle afferent sensory input to the maintenance of balance, the deficits we observe could contribute to balance instability during obesity.

### Sex differences

While the same general pattern of decreased muscle spindle afferent sensitivity in obesity was observed in both sexes during ramp and hold stretch, the effect was not as strong in the female mice for vibration sensitivity (Fig 3G & 3H) and baseline firing changes. This could be due to the decreased weight gain in females (Fig 2) and/or the fact that the metabolic and inflammatory responses to diet induced obesity are reduced in female mice [36, 49]. However, there was also a sex difference in muscle spindle afferent function in control conditions. To our knowledge this is the first study to directly compare male and female muscle spindle afferent responses, although mixed sex groups have been used in previous studies. Afferents from female mice had lower firing rates during the plateau phase of stretch (Fig 3C and 3D), were more likely to have lower or no firing at L_o_, and were less likely to entrain to vibration (Fig 3G & 3I). However, there was no sex difference in dynamic stretch measures (DP & DI; Fig 3E and 3F). Baseline, peak, and plateau tension normalized to CSA as well as both parallel and series muscle elasticity were lower in female mice (Table 1), which is consistent with the lower afferent firing rates observed during the static phase of stretch. Whether these sex differences in passive signaling properties occur in the whole animal or if they are compensated for with increased gamma motor neuron drive or other central nervous system mechanisms is unknown and should be addressed in future studies.

### Potential mechanisms for impaired proprioceptor function during obesity

Changes to muscle spindle afferent signaling could be due to changes in neural mechanosensation or changes in muscle mechanical properties. We did not observe any changes in muscle weight, CSA, L_o_, or maximal tetanic contractile force during high fat feeding, similar to previous findings [50, 51]. Both parallel and series muscle elasticity were also unchanged with diet induced obesity (Table 1). In short, we found no evidence of any muscle changes that could alter the mechanical forces seen by the muscle spindle afferents. However, the intrafusal fiber type(s) that muscle spindle afferents contact are critical for determining their sensitivity to muscle stretch and movement [25] and our measurements were not made on intrafusal fibers directly. Changes in the number and fiber composition of intrafusal fibers have been observed in other conditions, including aging [52, 53]. Future studies are needed to determine if spindle intrafusal fiber properties are altered with diet induced obesity.

Changes in plantar mechanoreceptor function are thought to be due to the increased weight overloading the sensitivity of the receptor [21] because when increased weight is added to a lean control a similar loss of plantar mechanoreceptor sensitivity is observed [54, 55]. Increased weight could potentially contribute to the observed sensory deficits in muscle spindle afferents as well, although we note that female mice showed the same deficits even though they gained much less weight than male mice (Fig 2). Interestingly, balance and gait deficits, including poor performance on the balance beam, are present in mice fed a HFD as well as mice fed a restricted amount of a HFD so that they don’t gain more weight than the CON mice [32]. This suggests that if the balance and muscle spindle afferent deficits share the same causal factor, it might be something other than weight gain alone.

In addition to weight gain, diet induced obesity is accompanied by metabolic changes, a chronic inflammatory state [56], and increased sympathetic nervous system activation [57]. These inflammatory and metabolic changes during obesity are also thought to contribute to changes in neural function, including cognitive decline and peripheral neuropathy [58], and could contribute to the changes in muscle spindle afferent response properties that we have observed. For instance, inflammatory factors enhance the response of nociceptive afferents to stimuli [59] and potentially alter muscle spindle afferent response properties as well since the activity of both ion channels shown to be involved in spindle afferent mechanotransduction, Piezo2 [60] and ASIC3 [61], can be modulated by inflammatory factors [62–64]. A HFD causes male, but not female mice, to exhibit low grade systemic and adipose tissue inflammation [36, 65]. Similarly 14 weeks on a HFD leads to the early stage of metabolic syndrome in male but not female mice, including hyperinsulinemia, higher blood glucose, and insulin tolerance [36]. Type II Diabetes is not observed and 16 weeks on a similar HFD does not lead to any nerve fiber loss or axon atrophy, suggesting that peripheral neuropathy is not contributing to our results [66]. Female mice still exhibited decreased muscle spindle afferent responsiveness following high fat feeding even though they are known to be protected from inflammation and experience less severe metabolic complications [36], which suggests that those factors are not likely to be causal in females. The muscle spindle capsule is innervated by sympathetic neurons [67] and in animal models sympathetic activation decreases the muscle spindle afferent response to stretch [68–71]. Future studies should test whether abnormal sympathetic activity, chronic inflammation, metabolic complications, and/or some other obesity-related effect contribute to decreased responsiveness of the muscle proprioceptors during high fat feeding.

### Limitations

In this study we measured mechanosensation in mouse muscle spindle afferents *in vitro* following diet induced obesity. We found lowered sensitivity to stretch that could contribute to changes in muscle tone, motor behavior, and/or balance if not compensated with increased spinal cord excitability or gamma motor neuron tone. We did not perform behavioral analyses on our mice to confirm gait and balance disturbances. However, we think it is likely that our mice did exhibit balance instability as other labs have seen both gait and balance disturbances at earlier time points and on a less severe HFD than we used. For instance, following 5 weeks on a 45% kcal fat diet, decreased rotorod performance and an increased number of slips on the balance beam were observed [32]. Concurrent balance and muscle spindle afferent changes are only suggestive of causality, though. Future studies should focus on determining the cause of reduced muscle spindle afferent sensitivity and whether normalizing function in the presence of obesity improves balance and motor function. Alternatively, the changes in muscle spindle afferent function we observed could be small enough that they do not lead to behavioral changes. If so, our results still provide insight into the basic biology of muscle spindle afferents and suggest a novel condition during which signaling can be altered.

### Summary and significance

Our study is the first to measure muscle spindle afferent function during obesity. We have shown changes in mechanosensation by muscle spindle afferents following diet induced obesity in adult mice of both sexes. Understanding why muscle spindle afferent function is altered by obesity may improve our understanding of how muscle spindle afferent function is regulated and suggest other conditions in which function may be altered. Muscle spindle afferent input is necessary for proprioception and motor control, and dysfunction to this system could contribute to the balance and gait dysfunction seen following diet induced obesity. While mice and other quadrupeds have different balance and motor control challenges than humans, mouse muscle spindle afferent responses to muscle movement are qualitatively similar to those seen in cats, rats, and humans [35, 72] and mice show similar metabolic and inflammatory responses to diet induced obesity [36]. Future studies should investigate whether obesity leads to deficits in muscle spindle afferent signaling in humans and contribute to the impaired balance and increased risk of falling seen in populations with obesity.

## Supporting information

S1 File Manuscript data file. All individual values used to prepare figures, table, and reported averages found in this file.
(XLSX)
